# InGaP electron spectrometer for high temperature environments

**DOI:** 10.1038/s41598-019-47531-8

**Published:** 2019-07-31

**Authors:** S. Butera, G. Lioliou, S. Zhao, M. D. C. Whitaker, A. B. Krysa, A. M. Barnett

**Affiliations:** 1 Space Research Group, School of Engineering and Informatics, University of Sussex, Brighton, BN1 9QT UK; 2 EPSRC National Epitaxy Facility, University of Sheffield, Mappin Street, Sheffield, S1 3JD UK

**Keywords:** Astronomical instrumentation, Electronic devices

## Abstract

In this work, a 200 μm diameter InGaP (GaInP) p^+^-i-n^+^ mesa photodiode was studied across the temperature range 100 °C to 20 °C for the development of a temperature-tolerant electron spectrometer. The depletion layer thickness of the InGaP device was 5 μm. The performance of the InGaP detector was analysed under dark conditions and then under the illumination of a 183 MBq ^63^Ni radioisotope beta particle source. The InGaP photodiode was connected to a custom-made low-noise charge-sensitive preamplifier to realise a particle counting electron spectrometer. Beta spectra were collected at temperatures up to 100 °C with the InGaP device reverse biased at 5 V. The spectrum accumulated at 20 °C was compared with the spectrum predicted using Monte Carlo simulations; good agreement was found between the predicted and experimental spectra. The work is of importance for the development of electron spectrometers that can be used for planetary and space science missions to environments of high temperature or extreme radiation (e.g. Mercury, Jupiter’s moon Europa, near-Sun comets), as well as terrestrial applications.

## Introduction

The ability to determine the energy of individual electrons and the number of the electrons detected at each particular energy plays a crucial role in space science. Electron spectrometers can be essential for understanding the composition, structure, and evolution of planets, moons, and comets. For example, planetary electron spectroscopy can give information about the interaction between the solar wind and planetary atmospheres and magnetospheres^1,2^. Such interactions are responsible, for example, for the polar aurorae observed at the Earth as well as at other planets. Correspondingly, the study of planetary electron environments can also help to improve understanding of the associated magnetospheres themselves^3,4^. The temperatures in such environments can be so high (e.g. 400 °C at Mercury^5^) as to make the use of conventional silicon electronics difficult. Furthermore, and of particular contemporary relevance, electron spectrometers may play an important role in the study of Jupiter’s moon Europa which has received research attention because of an extensive water ocean beneath its icy surface^6^. Because of the Jovian magnetosphere, Europa’s icy surface is bombarded with charged particles (predominantly electrons) resulting in potentially significant radiolytically-driven processes in the surface ice^7^. Indeed, compounds (e.g. SO_2_, CO_2_, and hydrated compounds) have been observed on the surface which are likely to be a result of radiolysis, at least in part^8–10^. However, observations of water vapour plumes venting from Europa^11–13^ have reinforced the possibility of a present-day surface-ocean linkage. Thus, it is possible that the surface chemistry is not solely a result of radiolysis but may also be a result of material from the ocean which has been deposited on the surface. A better understanding of Europa’s electron radiation environment is necessary to improve understanding of the relative roles played by radiolysis and the ocean in creating the observed surface chemistry. Furthermore, in understanding the surface chemistry, it may be possible to better establish the chemistry of the ocean, which has important implications for its habitability. Whilst the temperature at Europa (~ −140 °C^7^) is much lower than at Mercury, the radiation environment is extreme and damaging to conventional Si electronics. As such, there is impetus to develop instrumentation which uses radiation hard materials because it would increase the useful life of future Europa missions. Electron spectrometers can also be useful for studying electron driven radiolytic processes in comets^14^. Studying how electrons alter the chemical and physical properties of the cometary ices, synthesizing new molecules and destroying others, can help to determine comets’ structures, as well as give information about their origins. The temperatures at comets can also be high (e.g. 87 °C for Halley’s comet at 0.8 AU^15^) and thus there is interest in high temperature tolerant electron spectrometers for this application as well.

To date, a variety of different electron spectrometers have been developed and included on many planetary science spacecraft. The Jupiter Energetic Particle Detector Instruments (JEDI) on board JUNO has the aim of characterising and understanding Jupiter’s polar regions and aurorae^16^. The Electron Spectrometer (ELS) in the Cassini Plasma Spectrometer (CAPS)^17^ measured electron energies and velocities at Saturn, thus providing information about Saturn’s magnetosphere, as well as the ionization and chemical processes taking place at Titan and elsewhere. An electron spectrometer, the Pluto Energetic Particle Spectrometer Science Investigation (PEPSSI)^18^, was also part of the New Horizons mission to Pluto, where it studied solar wind interactions with Pluto’s atmosphere. Messenger’s Energetic Particle Spectrometer (EPS)^3^ analysed the energy, angular, and compositional distributions of electrons at Mercury, and BepiColombo’s Mercury Magnetospheric Orbiter (MMO)^19^ has the Mercury Electron Analyser (MEA) that will investigate plasma processes inside Mercury’s magnetosphere.

Wide bandgap semiconductor photodiodes coupled to low-noise preamplifier electronics are an alternative and desirable technology for developing radiation-hard direct detection electron spectrometers which have excellent energy resolution. Direct detection electron spectroscopy with wide bandgap photodiodes and suitable low-noise readout electronics has been demonstrated at room temperature using GaAs^20,21^, AlGaAs^22^, and SiC^23^ detectors. A GaAs electron spectrometer was subsequently developed for operation in the temperature range 100 °C to 20 °C with a view to production of a multi-mission capable electron spectrometer for space science^24^. Photomultiplier tubes^25^ and Langmuir probes^26^ are common alternatives to photodiodes for electron spectrometers. However, in many instances, direct detection semiconductor spectrometers have distinct advantages, for example, they are lower in mass, volume, and power consumption. However, at present, this can come at the cost of smaller active areas.

Recently, the III-V ternary compound In_0.5_Ga_0.5_P (bandgap = 1.9 eV at room temperature^27–29^) has been investigated as a detector material for photon counting X-ray spectroscopy. Photon counting spectroscopic X-ray photodiodes made of In_0.5_Ga_0.5_P have been reported working at room temperature^30^ and at up to 100 °C^31^, with energy resolutions (Full Width at Half Maximum) at 5.9 keV of 840 eV and 1.27 keV at 20 °C and 100 °C, respectively. Table 1 compares the energy resolution at 5.9 keV of X-ray spectrometers made of competitive semiconductors^32–35^. At room temperature, the energy resolution at 5.9 keV of In_0.5_Ga_0.5_P is higher than that of AlInP, but worse than those of Si, GaAs, and SiC; at 100 °C, the only material that shows smaller FWHM at 5.9 keV is SiC. It should be noted that the good energy resolution at 5.9 keV observed for the SiC detector is due to the very low-noise readout electronics used in this case. Although Si presents a good FWHM at room temperature, it is not commonly used at 100 °C (without cooling) due to its significantly degraded performance.

**Table 1:** Energy resolution (FWHM) at 5.9 keV for Si, GaAs, AlInP, and SiC detectors at different temperatures.

Material	FWHM @ 5.9 keV
Si^32^	141 eV at 21 °C
GaAs^33^	690 eV at 20 °C 2 keV at 100 °C
AlInP^34^	930 eV at 20 °C 1.57 keV at 100 °C
SiC^35^	196 eV at 30 °C 233 eV at 100 °C

The use of InGaP for photon counting spectrometers is relatively new; only recently has their suitability for photon counting X-ray spectrometers at 20 °C^30^ and above^31^ been reported in the literature; temperature tolerant In_0.5_Ga_0.5_P spectrometers are interesting, since GaP and InP (InGaP binary relations) were found to not be spectroscopic even at 20 °C. The advantages of InGaP include radiation hardness, temperature tolerance, and higher linear absorption coefficients. InGaP has been proven to be radiation resistant to alpha particles and electrons, it has been successfully used for the development of alpha-voltaic (under the illumination of ^241^Am and ^210^Po alpha-particle radioactive sources^36^) and beta-voltaic microbatteries (under the illumination of ^3^H radioactive source^37^). The InGaP higher linear absorption coefficients results in a higher percentage of energy absorbed per unit thickness compared to those of other wide bandgap materials, including GaAs, AlGaAs, SiC, and AlInP. Moreover, In_0.5_Ga_0.5_P can be grown with high crystalline quality nearly lattice matched with GaAs substrates, thus making commercial production relatively simple.

In this article, the first use of an InGaP photodiode for direct detection particle counting electron spectroscopy is reported. A circular In_0.5_Ga_0.5_P p^+^-i-n^+^ mesa photodiode (200 μm diameter) (depletion layer thickness 5 μm) was coupled to a custom-made low-noise charge-sensitive preamplifier and studied over the temperature range 100 °C to 20 °C. The performance of the In_0.5_Ga_0.5_P electron spectrometer was analysed under the illumination of a 183 MBq ^63^Ni radioisotope beta particle source. The spectrum accumulated at 20 °C was then compared with the spectrum expected using Monte Carlo simulations which took into account the detectors’ layer structure and other aspects of the experimental set up.

## Device Structure

The details of the In_0.5_Ga_0.5_P p^+^-i-n^+^ photodiode used for the direct detection of electrons are presented in Table 2; more information about the growth and fabrication processes can be found in the Method section.

**Table 2:** Layer details of the In_0.5_Ga_0.5_P photodiode.

Layer	Material	Thickness (μm)	Dopant	Dopant Type	Doping density (cm^−3^)
1	GaAs	0.01	Zn	p^+^	1 × 10^19^
2	In_0.5_Ga_0.5_P	0.2	Zn	p^+^	2 × 10^18^
3	In_0.5_Ga_0.5_P	5	undoped		<10^16^
4	In_0.5_Ga_0.5_P	0.1	Si	n^+^	2 × 10^18^
5	GaAs buffer	0.3	Si	n^+^	2 × 10^18^
6	Substrate n^+^ GaAs	350	Si	n^+^	2 × 10^18^

Relatively soft energy X-rays and relatively soft energy electrons have similar interaction mechanisms with a semiconductor material. In a semiconductor X-rays/electron spectrometer, an X-ray/electron interacts with the semiconductor, depositing all or part of its energy so that electron-hole pairs are generated (the average number of electron-hole pairs generated depends on the semiconductor electron-hole pair creation energy and the amount of energy deposited). X-rays and electrons are expected to be absorbed within the In_0.5_Ga_0.5_P i region: each of the X-rays can be considered to be absorbed in one location of the i layer; whilst each electron loses energy along its trajectory through the i layer. Because of the combination of built in and applied electric field, the electron-hole pairs in the depletion region are swept to the detector electrodes (electrons move toward the cathode; whilst holes toward the anode); the movement of these generated carriers induces a charge on the contact of the semiconductor photodiode and produced a current pulse. The induced charge on the electrodes can be calculated by the Shockley-Ramo theorem^38,39^. Incomplete charge collection due to charge trapping and recombination can degrade the spectrometer’s energy resolution. Other sources of noise that degrade the spectrometer’s energy resolution are Fano noise (which takes into account the statistical nature of the charge creation processes) and electronic noise which depends on the electrical characteristics of detector and readout electronics.

## Experimental Results

### Current and capacitance measurements

The dark current characteristic of the In_0.5_Ga_0.5_P photodiode was studied as functions of reverse bias using a Keysight B2981A femtoammeter/picoammeter. A Keithley 2636B source meter was used to apply the bias to the photodiode; the reverse bias on the In_0.5_Ga_0.5_P detector was increased from 0 V to 30 V in 1 V steps. Using a TAS Micro MT climatic cabinet, the current of the photodiode was studied over the temperature range 100 °C to 20 °C (in 20 °C steps). At each temperature, before taking any measurements, the device was left for 30 minutes to ensure thermal equilibrium and stabilisation. Dry N_2_ was used to decrease the relative humidity inside the climatic cabinet to less than 5% in order to prevent any effects due to water vapour at high temperatures or water condensation at low temperatures, which can critically affect the performance of the not-hermetically sealed TO-5 can where the In_0.5_Ga_0.5_P device was packaged (e.g. leakage current).

**Figure 1:**
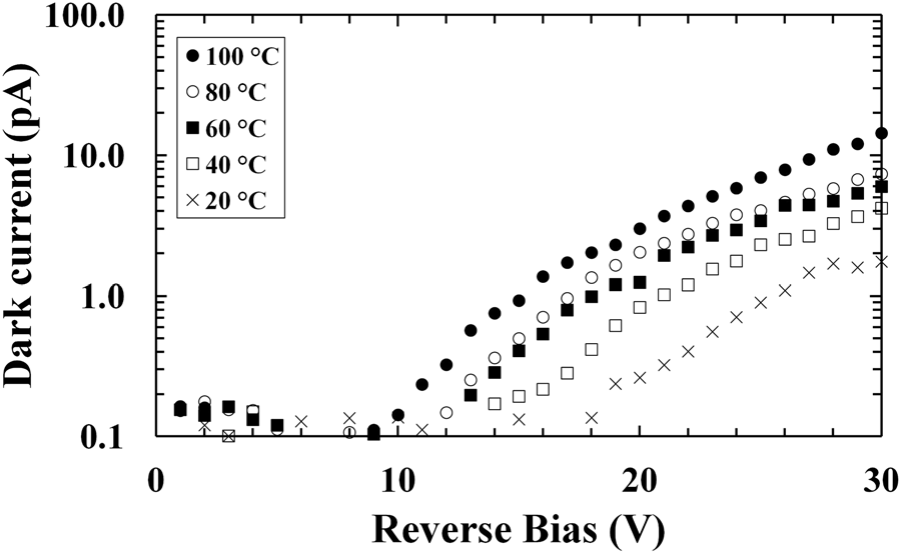
Dark current of the In_0.5_Ga_0.5_P photodiode itself (dark current from the package subtracted) as a function of applied reverse bias at 100 °C (filled circles), 80 °C (empty circles), 60 °C (filled squares), 40 °C (empty squares), and 20 °C (crosses).

Firstly, the current of the In_0.5_Ga_0.5_P device was analysed in dark condition; the measured current has contributions from both the In_0.5_Ga_0.5_P semiconductor junction and the imperfect insulators of the TO-5 can, in which the diode was packaged. Such dark current was slightly higher than that observed in ref.^30^ (at 30 V and 20 °C, 2 pA against 0.5 pA) and ref.^31^ (at 15 V and 100 °C, 4 pA against 1.5 pA), where similar devices were studied. The observed increase was within the experimental measurement repeatability accuracy. Figure 1 shows the leakage current contribution from the In_0.5_Ga_0.5_P photodiode itself (calculated as explained in the Method section). As expected, the dark currents of the photodiode were smaller at lower temperatures.

**Figure 2:**
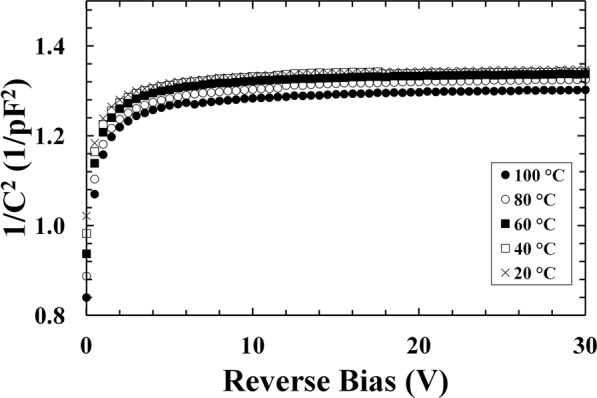
1/*C*^2^ as a function of applied reverse bias for the In_0.5_Ga_0.5_P detector at 100 °C (filled circles), 80 °C (empty circles), 60 °C (filled squares), 40 °C (empty squares), and 20 °C (crosses).

In the same range of temperatures, the capacitance of the In_0.5_Ga_0.5_P detector was measured as a function of reverse bias using an HP 4275 A Multi Frequency LCR meter and a Keithley 6487 Picoammeter/Voltage Source as the external voltage supply. The test signal was sinusoidal with a 50 mV rms magnitude and 1 MHz frequency. Reverse biases from 0 V to 30 V were applied to the photodiode (in 1 V steps). As for the current measurements, dry N_2_ was used to achieve a relative humidity <5%. The measured capacitance had a contribution from the In_0.5_Ga_0.5_P device itself as well as from the TO-5 can, where the device was packaged. The latter is defined as capacitance of the empty package (no diode attached). Knowing the capacitance of the In_0.5_Ga_0.5_P detector itself (estimated as explained in the Method section) is important for calculating its depletion layer as well as doping concentrations within the structure. Such capacitance was found to decrease when the temperature was reduced from 100 °C to 20 °C, particularly at low reverse biases. The capacitance (depletion width) increased (decreased) with increasing temperature possibly because of progressive ionization of dopants at high temperature. At low temperatures, a thin region of non-ionized dopants may be present around the depletion layer; increasing the temperature, the dopants in such region progressively ionized thus increasing the capacitance of the device (decreasing the extension of the depletion layer)^33,40^. A dependence between the capacitance and the bias was found at reverse biases <5 V; the capacitance was instead constant at reverse biases >5 V. In Fig. 2, 1/*C*^2^ as a function of reverse bias from 100 °C to 20 °C is shown.

The capacitance and the leakage current results reported in this section are extremely important, since they affected the energy resolution (FWHM) of the In_0.5_Ga_0.5_P spectrometer^41^.

### Beta spectroscopy experiment

The photodiode was then connected to a custom-made, single channel, charge-sensitive preamplifier of feedback resistorless design^42^. The output of the preamplifier was connected to an Ortec 572A shaping amplifier which was connected to an Ortec Easy 8K multichannel analyser (MCA). The Ortec 572A shaping amplifier had a Semi-Gaussian pulse shape, a gain of 71.85 (set by the Authors), pole-zero cancellation, and baseline restore discriminator^43^.

After the interaction of an electron from the beta radioisotope source with the semiconductor, a charge pulse is produced by the detector. Such pulse is then processed by the custom-made low-noise charge-sensitive preamplifier, the shaping amplifier, and the MCA. The shaping amplifier converts the preamplifier’s output signal into a form more suitable for the MCA. The shaping amplifier has six switch-selectable shaping times to provide optimum shaping for resolution and count rate. The first differentiation network has variable pole-zero cancellation; the pole-zero cancellation drastically reduces the undershoot after the first differentiator and greatly improves overload and count rate characteristics. In addition, the shaping amplifier contains an active filter shaping network that optimises the signal-to-noise ratio and minimises the overall resolving time. A baseline restorer circuit is also included for improved performance at all count rate^43^. The MCA used consists of a 13 bit analogue digital convertor to convert pulse height into a digital number which is added into an 8k histogram by an onboard FPGA^44^. Since an external triggering scheme has not been used in the beta spectroscopy experiment, the total deposited energy from a single beta particle within the In_0.5_Ga_0.5_P active layer corresponded to a single event in the histogram at the channel number associated to that energy.

**Figure 3:**
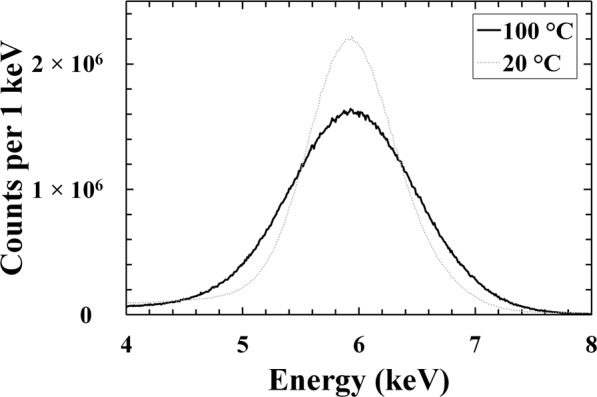
^55^Fe X-ray spectra accumulated at 5 V reverse bias using the In_0.5_Ga_0.5_P device under the illumination of ^55^Fe radioisotope X-ray source at 100 °C (black solid line), and 20 °C (grey dashed-dotted line). In the spectra the number of counts per 1 keV has been shown to take into account the different widths of the channels at different temperatures.

The In_0.5_Ga_0.5_P photodiode and preamplifier assembly was installed inside the TAS Micro MT climatic cabinet for temperature control; dry N_2_ was constantly flowing inside the cabinet to maintain a relative humidity <5%. Prior to accumulation of the beta particle spectra, the detector of the spectrometer was first illuminated with a 167 MBq ^55^Fe radioisotope X-ray source (Mn Kα = 5.9 keV, Mn Kβ = 6.49 keV), which had an active surface of 6 mm in diameter. Figure 3 shows the ^55^Fe spectra accumulated at 100 °C and 20 °C, using optimum shaping times as reported in ref.^31^.

The number of electron-hole pair generated (*N*) in the In_0.5_Ga_0.5_P photodiode after the absorption of a photon of energy *E* is given by *N* = *E*/*ω*, where *ω* is the In_0.5_Ga_0.5_P electron-hole pair creation energy; at 20 °C, *ω* = 4.94 eV^31^. The spectrometer required 2 keV of energy to be deposited within its active layer in order for it to detect the electron. The 2 keV threshold was set to eliminate any contribution from the spectrometer’s zero energy noise peak; thus, any events below this energy could not be detected by the spectrometer. In the temperature range studied, the average count rate within the 5.9 keV peak was 2200 s^−1^. It should be noted that with the chosen activity of the ^55^Fe radioisotope source, In_0.5_Ga_0.5_P device area, and source/device distance, pulse pile up effects were negligible. The dead time of the spectroscopic system increased from 5% to 11% between 100 °C and 20 °C. The measured FWHM at 5.9 keV of the In_0.5_Ga_0.5_P photodiode varied from 1.23 keV (104 e^−^ rms) at 100 °C (given an electron-hole pair creation energy of 5.02 eV^31^) to 850 eV (73 e^−^ rms) at 20 °C (given an electron-hole pair creation energy of 4.94 eV^31^), similar to ref.^31^. The Auger electrons from the ^55^Fe radioisotope source did not affect these measurements as they were stopped by the radioisotope X-ray source’s Be window (0.25 mm thick).

The energy resolution of an X-ray photon/particle counting spectrometer is broadened due to the Fano noise (which takes into account the statistical nature of the charge creation process), electronic noise, and incomplete charge collection noise^41^. At 5.9 keV the Fano noise was estimated to be 139 eV FWHM (12 e^−^ rms) at 20 °C given an electron-hole pair creation energy of 4.94 eV^31^ and assuming a Fano Factor of 0.12. The electronic noise includes series white noise, parallel white noise, induced gate current noise, 1/*f* noise, and dielectric noise. The series white noise is given by Eq. 1 ^41^:

1 $$EN{C}_{ws}=\frac{B}{q}\sqrt{\frac{{A}_{1}}{2}4kT\frac{\gamma }{{g}_{m}}{({C}_{T})}^{2}\frac{1}{\tau }}$$

where *B* is the induced gate current correction (0.8), *A*_1_ is 1.85, γ is the product of the noise resistance and the transcoductance of the Si JFET, *g*_*m*_ is the tranconductance of the Si JFET, and *C*_*T*_ is the total capacitance at the preamplifier input (= *C*_*d*_ + *C*_*i*_ + *C*_*f*_ + *C*_*s*_, where *C*_*d*_ is the detector capacitance, *C*_*i*_ is the input Si JFET capacitance, *C*_*f*_ is the feedback capacitance, and *C*_*s*_ is the stray capacitance)^41^.

The parallel white noise is given by Eq. 2^41^:

2 $$EN{C}_{wp}=\frac{1}{q}\sqrt{\frac{{A}_{3}}{2}2q({I}_{d}+{I}_{i})\tau }$$

where *A*_3_ is 1.85, *I*_*d*_ is the leakage currents of the detector, and *I*_*i*_ is the leakage current of the input Si JFET of the preamplifier^41^.

At 20 °C and using a shaping time *τ* = 10 *μ*s, the known series white noise was 8 e^−^ rms (this was only due to the capacitances of the photodiode and the input Si JFET; the Si JFET capacitance was assumed to be 2 pF). At the same temperature and shaping time a parallel white noise of 11 e^−^ rms was estimated (the Si JFET leakage current was assumed to be 1 pA). At 20 °C, a known 1/*f* noise of 2 e^−^ rms and a known dielectric noise of 53 e^−^ rms (using dielectric dissipation factors of 4.2 × 10^−3^ for In_0.5_Ga_0.5_P^30^ and 2 × 10^−3^ for Si^45^) were calculated, these noise contributions are shaping time independent. It should be noted that a direct measurement of the Si JFET leakage current and capacitance could not be performed, the values used in the calculation are based on the Si JFET datasheet^46^.

At each temperature, the spectrometer’s charge scale was energy calibrated using the characteristic Mn Kα (5.9 keV) emissions from the ^55^Fe radioisotope X-ray source and the spectrometer’s zero energy noise peak. The 183 MBq ^63^Ni radioisotope beta particle source (end point energy of 66 keV; active surface of 7 mm × 7 mm) was then used to investigate the beta response of the spectrometer. To comply with laboratory safety protocols, the ^63^Ni radioisotope beta source had a protective inactive Ni over-layer (1 μm thick). Both radioactive sources were placed ~3 mm away from the detector top surfaces. In each case the In_0.5_Ga_0.5_P photodiode was reverse biased at 5 V (this bias was chosen in accordance with the capacitance results, Fig. 2, since the diode was fully depleted at 5 V).

**Figure 4:**
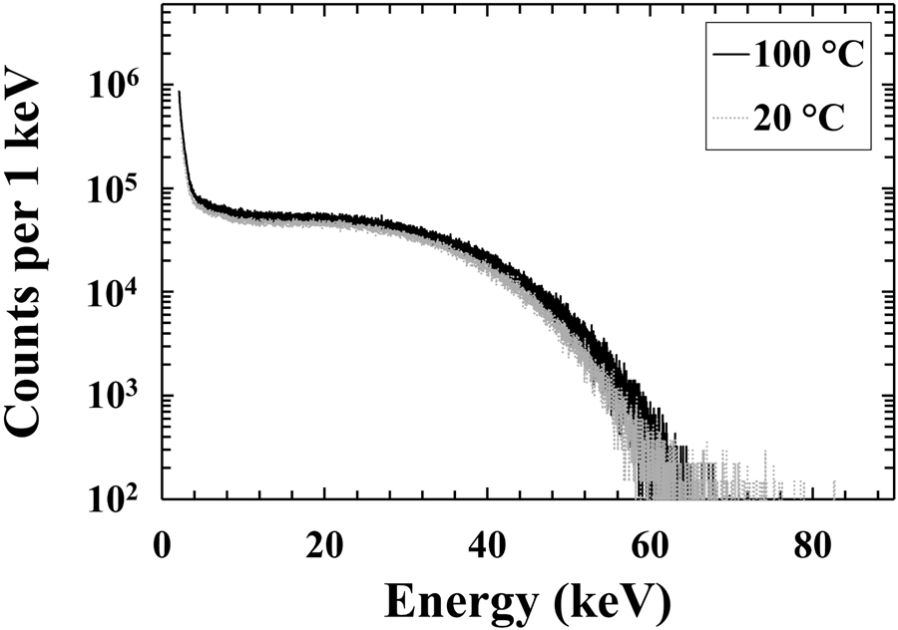
^63^Ni beta spectra accumulated at 5 V reverse bias using the In_0.5_Ga_0.5_P device under the illumination of ^63^Ni radioisotope beta source at 100 °C (black solid line), and 20 °C (grey dashed-dotted line). In the spectra the number of counts has been normalised to take into account the different widths of the channels at different temperatures. Because the spectra have been standardised into counts per 1 keV to take account of the differing channel widths in the raw data, the minimum non-zero number of counts in each spectrum’s channels was equivalent to 100 per keV.

The accumulation time for each beta spectrum was 1800 s; an optimum shaping time was used at each temperature. The optimum shaping time used for the each beta spectrum was determined from preliminary results obtained under the illumination of the ^55^Fe radioisotope X-ray source^31^. At each temperature, the shaping time was chosen to be that at which the best energy resolution (Full Width at Half Maximum at 5.9 keV) was observed during the ^55^Fe X-ray measurements. The optimum shaping time was 1 μs from 100 °C to 60 °C, and 10 μs at temperatures ≤40 °C. The beta spectra obtained at 100 °C and 20 °C are shown in Fig. 4; the average count rate at electron energies above 2 keV were 1300 s^−1^ at 100 °C and 1000 s^−1^ at 20 °C. Comparable spectra were obtained for the other temperatures as well, but they are omitted from the figure for clarity.

At low energies (<8 keV), an increased number of counts was observed beyond that which could be explained directly by the statistics of the beta decay of ^63^Ni^47^. The increased number of counts at low energy cannot be attributed to the zero-energy noise peak of the preamplifier assuming the characteristics of the zero-energy noise peak were the same in beta-illuminated condition as they were in unilluminated and X-ray illuminated condition. The zero-energy noise peak was entirely eliminated by an MCA low energy discriminator of ~2 keV in the latter (X-ray) case. One possibility is that the beta illumination increased the stead-state component of the detector current thus broadening the zero-energy noise peak. A similar phenomenon with a possibly different aetiology was observed in a GaAs spectrometer illuminated with a ^14^C radioisotope beta particle source^21^; in that case the increased number of counts at low energies was attributed to increased series white noise as a consequence of additional stray capacitance brought by the radioisotope beta particle source adding additional capacitive load to the preamplifier’s input. However, that explanation cannot apply to the present case because the front-end of the preamplifier was well controlled. Nevertheless, the spectra indicate that the spectrometer is spectroscopic for beta particles of energy ≈8 keV and greater, and the response is broadly consistent across the temperature range. Whilst the end point energy of ^63^Ni is 66 keV, the end point detected by the spectrometer was ≈62 keV. The counts beyond 62 keV (up to ≈80 keV) were mainly attributed to pulse pile up phenomena within the device, with some small contributions from the energy resolution of the detector at 62 keV. The low counts beyond 62 keV, however, suggested insignificant pulse pile up. The lower end point energy is explained by the Monte Carlo simulations discussed below.

### Monte carlo simulations

The beta spectrum expected to be detected by the In_0.5_Ga_0.5_P device under the illumination of the ^63^Ni radioisotope beta particle source was predicted through the use of the Monte Carlo computer modeling package CASINO (version 2.4.8.1)^48,49^. This program allowed the study of the beta particles’ interactions with the protective inactive Ni over-layer of the radioisotope beta particle source, with the dry N_2_ atmosphere between the detector and radioisotope beta particle source, and with the In_0.5_Ga_0.5_P structure itself.

**Figure 5:**
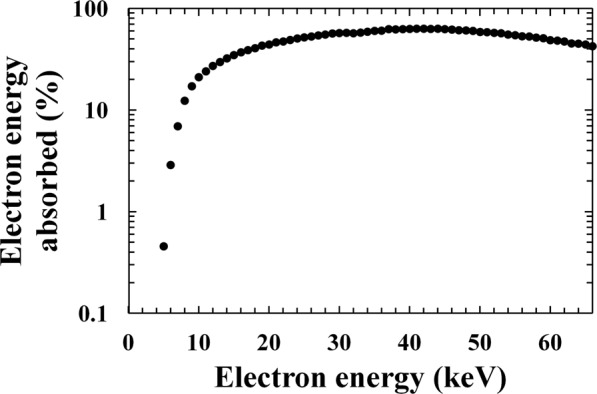
Percentage of electron energy absorbed in the i layer of the In_0.5_Ga_0.5_P photodiode as a function of electron energy, as determined by Monte Carlo modelling using CASINO.

Firstly, the percentage of electron energy absorbed in the i layer of the In_0.5_Ga_0.5_P detector was studied in the energy range 1 keV to 66 keV (^63^Ni endpoint energy) in 1 keV steps. The Monte Carlo simulation used tabulated Mott elastic cross sections and experimentally determined stopping powers to estimate the trajectory of each electron within the detector, and compute each electron’s energy at each trajectory position. The percentage of each electron energy absorbed in the i layer was calculated by the ratio between the electrons’ energy absorbed in the 5 μm i layer In_0.5_Ga_0.5_P device and the electrons’ energy incident on the detector’s face. Since the top Ohmic contact covered 45% of the In_0.5_Ga_0.5_P top surface, simulations were carried with electrons incident on the contact and incident on the area not covered by the contact. The results were combined in the appropriate weights to reflect the areas of the photodiode covered and uncovered by the contact. In each simulation, 4000 beta particles per energy were simulated as incident on the top of the detector. In the calculations of the percentage of electron energy absorbed, only the photodiode’s 5 μm In_0.5_Ga_0.5_P i layer was considered to be active; energy deposited in other regions of the photodiode was considered to have been lost. Figure 5 shows the percentage of electron energy absorbed in the i layer of the In_0.5_Ga_0.5_P device as computed in this manner. It should be noted that this was a conservative “worst case” assumption which likely leads to an underestimate of the percentage of the electron energy absorbed at low energies, since it is probable that at least a portion of the detector’s p layer was also active.

Scattering of electrons on the detector’s surface and interaction of electrons with dead layers reduced the detected energies. Electrons with energy <4 keV which are incident on the area of the detector not covered by the metal contact were absorbed in the GaAs and p^+^ In_0.5_Ga_0.5_P dead layers, and did not reach the In_0.5_Ga_0.5_P intrinsic layer. Electrons with energy <10 keV which were incident on the area detector covered by the metal contacts did not reach the In_0.5_Ga_0.5_P intrinsic layer because they were absorbed in the Ti/Au metal contacts as well as GaAs and p^+^ In_0.5_Ga_0.5_P dead layers. At 62 keV (the detected end point energy) 47% of the electron energy was absorbed; by increasing the thickness of the In_0.5_Ga_0.5_P i layer to 40 µm, the percentage of electron energy absorbed would rise to 74% at 62 keV and to 80% for electrons of 100 keV. Such values were limited by the front dead layers of the In_0.5_Ga_0.5_P photodiode. The detection of energies up to 100 keV would be of interest, for example, for the study of the Europa electron environment; such soft electron energy range is very interesting because of its higher electron flux (the electron flux for such soft electrons is up to four order of magnitudes higher than the electron flux for harder electrons^7^).

**Figure 6:**
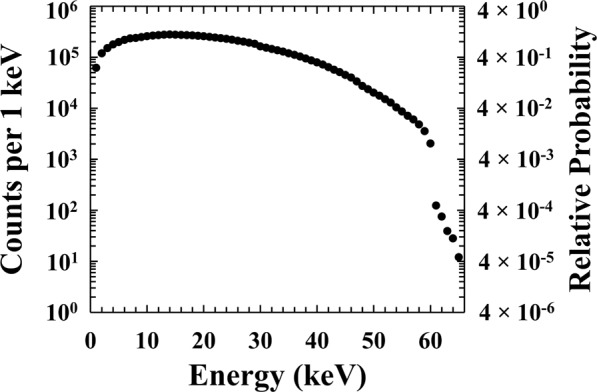
Simulated ^63^Ni energy spectrum incident upon the In_0.5_Ga_0.5_P detector’s surface calculated using Monte Carlo computer modelling package CASINO. It should be noted that the number of electrons simulated was chosen to provide good statistics across the energy range rather than to reflect directly the number of electrons emitted by the ^63^Ni radioisotope beta particle source during the acquisition of the experimental spectra (accumulation time of 1800 s).

Secondly, the attenuation of the beta electrons through the ^63^Ni radioisotope beta particle source’s protective inactive Ni over-layer (1 μm thick) and the dry N_2_ layer (3 mm thick) were estimated in the energy range 1 keV and 66 keV. The emitted energy spectrum of ^63^Ni^47^, taking into account self-absorption effects, was considered in order to simulate at each energy the appropriate relative number of electrons emitted from the 3 μm thick ^63^Ni region of the source. At 21 keV (the most probable beta particle energy from a ^63^Ni radioisotope beta particle source when self-absorption effects are included), 565500 electrons were simulated. The number of electrons simulated at other energies reflected the relative emissions probabilities of ^63^Ni. 18483562 electrons were simulated in total. The simulations were parallelized on a bank of 12 computers each with an Intel i7-6700 4 core 3.4 GHz processor and 32 GB of random access memory. Convergence studies showed that the number of particles simulated at each energy was sufficient for the statistics to be reflective of reality and suitable for comparison to the experimentally obtained equivalent. The number of electrons simulated was chosen for this reason, rather than to reflect directly the numbers of electrons emitted and detected. Thus, by considering losses through self-absorption of the source, losses in the inactive over-layer, and the dry N_2_ atmosphere, the beta particle spectrum incident on the detectors surface was computed, as shown in Fig. 6. Although beta particles with energies up to 66 keV are emitted from ^63^Ni, the simulations show that the relative number of beta particles incident on the detector which have energies greater than 60 keV is small due to energy losses along the electrons’ trajectories towards the detector.

**Figure 7:**
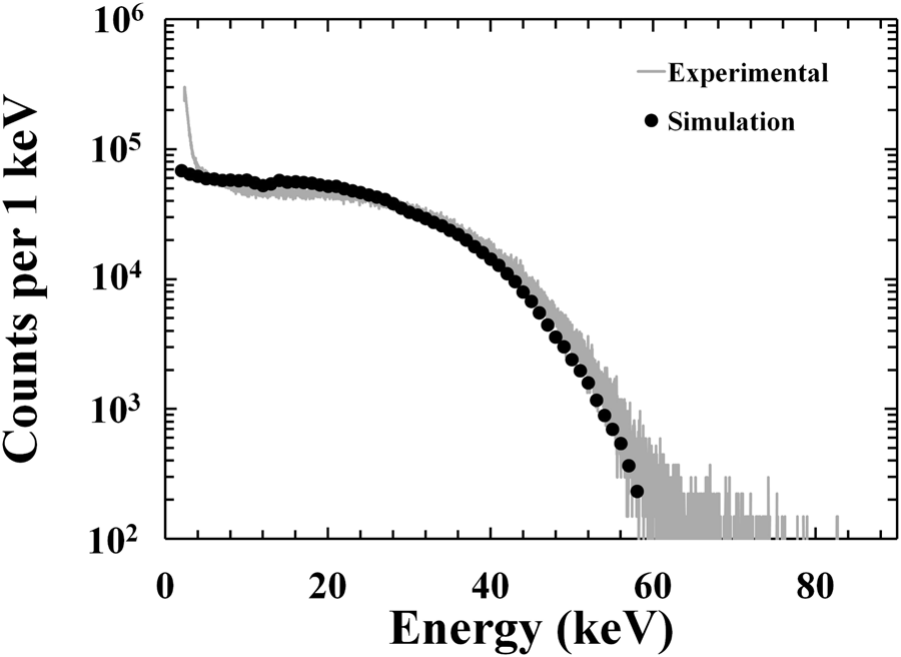
Comparison between the experimental ^63^Ni beta spectrum at 20 °C (grey line) and the spectrum predicted using Monte Carlo simulations (black dashed line).

By combining the spectrum shown in Fig. 6 with the percentage of the electron energy absorbed in the detector i layer as a function of energy (presented as Fig. 5), the spectrum expected to be detected by the spectrometer was computed (excluding Fano noise, electronic noise, detector side-edge effects, and pulse pile up). The results of the simulations are shown in Fig. 7, where they are qualitatively compared with the spectrum that was experimentally obtained at a temperature of 20 °C. For the comparison, the simulated spectrum was normalised to reflect the number of electrons emitted by the ^63^Ni radioisotope beta particle source during the accumulation time of 1800 s.

As shown in Fig. 7, the simulated and experimentally accumulated spectra were remarkably similar, even if the simulations did not take into consideration the spectrometer’s energy resolution, edge effects of the detector, and pulse pile up effects, which could account for the small discrepancy at high energy. Future theoretical models will include the possibility of all these phenomena within the device and possibly clarify the results obtained above 60 keV.

As shown in Figs 4 and 7, the In_0.5_Ga_0.5_P electron spectrometer can spectroscopically detect the electrons incident upon it from the ^63^Ni radioisotope beta particle source at temperatures up to 100 °C. This is the first time that an electron spectrometer with a detector made of this material has been reported.

## Discussion and Conclusion

In this paper, a particle counting In_0.5_Ga_0.5_P electron spectrometer has been demonstrated for the first time and shown to operate at temperatures up to 100 °C. A 200 μm diameter In_0.5_Ga_0.5_P photodiode (depletion layer thickness 5 μm) was used to measure the energy spectrum of the electrons emitted by a 183 MBq ^63^Ni radiosotope beta particle source. In_0.5_Ga_0.5_P photodiode electrical characterisation was performed at reverse biases up to 30 V. At 100 °C, dark currents ≤0.1 pA and <20 pA were observed at 5 V and 30 V, respectively. Beta spectra were collected with the In_0.5_Ga_0.5_P diode at 5 V reverse bias; similar spectra were measured at all the temperatures investigated, thus demonstrating that the spectrometer’s performance was relatively impervious to temperature changes over this range. Monte Carlo simulations were performed such to predict the expected detected spectrum; the results of the simulations were compared to the experimental spectrum accumulated at 20 °C; the simulations and experimental results were found to be in agreement.

Such electron spectrometers are important for future solar system science. For example, as discussed in the Introduction, measurements of the electron environment at Europa would help in understanding the radiolytic chemistry of its surface, and thus potentially advance knowledge of the extent of linkage between its ocean and surface. In turn, this may even enable inference of some properties of the Europan ocean. Furthermore, because of its ability to work at high temperatures, the In_0.5_Ga_0.5_P spectrometer is also advantageous for use in hot environment space missions. Temperatures of 100 °C (and even greater depending on latitude) can be found on the surface of Mercury^5,50^, where electron spectrometers of this type could be used to study the solar wind interactions with the planet and its magnetic field *in situ*. Study of comets is another potential application of such spectrometers due to the high temperatures reached at perihelion^15^. At comets, such instrumentation could be used in a role similar to that at Europa, namely to investigate the electron environment with a view to understanding electron induced radiolytic processes of cometary ices, and the potential resultant organic molecules.

## Methods

### Growth and fabrication processes

An In_0.5_Ga_0.5_P p^+^-i-n^+^ epilayer was grown using metalorganic vapour phase epitaxy (MOVPE) on a heavily doped (100) n^+^ GaAs substrate. In order to suppress CuPt type ordering and associated decrease of the bandgap energy of In_0.5_Ga_0.5_P^51–53^, the substrate’s epitaxial surface had a miscut angle of 10° towards <111 > A. The In_0.5_Ga_0.5_P epilayer consisted of a 0.2 μm p^+^ layer (doping concentration of 2 × 10^18^ cm^−3^), a 5 μm i layer, and a 0.1 μm n^+^ layer (doping concentration of 2 × 10^18^ cm^−3^). Zn and Si were used as the p type and n type dopant atoms. On top of the In_0.5_Ga_0.5_P epilayer, a 0.01 μm p^+^ GaAs layer (doping concentration of 1 × 10^19^ cm^−3^) was grown to facilitate the formation of a top Ohmic contact. The top Ohmic contact consisted of Ti/Au (20 nm/200 nm thick); whilst the bottom Ohmic contact consisted of InGe/Au (20 nm/200 nm thick). Device fabrication used standard photolithography and wet chemical etching solutions (1:1:1 K_2_Cr_2_O_7_:HBr:CH_3_COOH solution followed by a 10 s finishing etch in 1:8:80 H_2_SO_4_:H_2_O_2_:H_2_O solution). A 200 μm diameter In_0.5_Ga_0.5_P mesa device was fabricated, its mesa sidewalls were not passivated; the top Ohmic contact covered 45% of the device surface and had an annular shape. The In_0.5_Ga_0.5_P photodiode was packaged in a TO-5 can.

### Current and capacitance measurement technique

Because of the contribution from the TO-5 can to the measured leakage current and capacitance, dark current and capacitance measurements of an empty package (no diode connected) were made and subtracted from the measured dark current and capacitance values. This procedure allowed to evaluate the current and capacitance contribution from the In_0.5_Ga_0.5_P photodiode itself, as shown in Figs 1 and 2.

## Data Availability

Whilst all data from the study and the findings are contained within the paper, further requests for information may be addressed to the authors.
